# Attention-Enhanced Multi-Scale Feature-Wise Linear Modulation for Fine-Grained Poisonous Mushroom Image Recognition

**DOI:** 10.3390/jimaging12080398

**Published:** 2026-08-21

**Authors:** Yuan He, Haikun Lv, Chenyang Lu, Dengqi Yang, Xiaowei Li, Lina Zhang

**Affiliations:** School of Mathematics and Computer Science, Dali University, Dali 671003, China; hy2050@163.com (Y.H.); lvhk000704@163.com (H.L.); luucyok@163.com (C.L.); dengqiyang@163.com (D.Y.); lixiaowei_xidian@163.com (X.L.)

**Keywords:** mushroom recognition, fine-grained classification, feature modulation, dual-backbone network, interpretability, natural scenes

## Abstract

Fine-grained poisonous mushroom recognition in natural scenes is challenging because of complex backgrounds, subtle morphological differences, and the limited interpretability of model decisions. To address these challenges, this paper proposes Att-FiLM, an attention-enhanced multi-scale Feature-Wise Linear Modulation network for poisonous mushroom image recognition. The model adopts an asymmetric dual-backbone architecture in which a frozen ConvNeXt-Base branch provides global semantic priors, while a trainable EfficientNet-B0 branch learns local discriminative features. Rather than directly concatenating heterogeneous features, Att-FiLM generates scale and shift parameters from semantic features and performs channel-wise modulation on multi-scale EfficientNet features at Stage 2 and Stage 4. This mechanism enables global semantic information to guide local feature learning while reducing feature redundancy and semantic inconsistency. Experimental results show that Att-FiLM achieves an Accuracy of 95.58% and an F1-score of 0.9455 on the poisonous/edible binary classification task. On the 190-class species-level classification task, it achieves a Top-1 Accuracy of 93.63% and a Macro-F1 of 0.9347. Interpretability analysis further shows that decision-relevant responses are frequently associated with morphologically relevant regions, including gills, annuli, volvae, and cap textures. These results indicate that Att-FiLM provides effective recognition performance together with interpretable decision evidence for mushroom recognition in complex natural scenes.

## 1. Introduction

Wild mushroom poisoning remains a persistent public health and food safety concern, particularly in regions where wild mushroom collection and consumption are common. Poisonous and edible mushrooms may exhibit highly similar macroscopic appearances, including cap shape, gill structure, stipe texture, annulus, and volva morphology, making reliable identification difficult for non-experts [1,2,3]. Conventional identification generally relies on expert morphological examination, field guides, or laboratory analysis. Although these approaches can provide reliable results, they require specialized knowledge and are difficult to apply efficiently in natural outdoor environments [4].

Deep learning has provided new opportunities for automated mushroom image recognition. Nevertheless, recognition under natural-scene conditions remains challenging because the visual differences between closely related mushroom categories are often subtle and localized. Discriminative information may appear in small morphological regions, such as gills, annuli, volvae, or cap textures, while illumination changes, occlusion, soil, grass, and fallen leaves can introduce substantial background interference. These characteristics make poisonous mushroom recognition closely related to the fine-grained visual recognition problem, in which models must preserve local morphological details while maintaining sufficient global semantic context.

Existing deep visual models can extract strong image representations, but two limitations remain relevant to this task. First, a single backbone may gradually weaken fine local cues as features become increasingly semantic at deeper layers. Multi-scale or multi-branch architectures can alleviate this problem, but directly concatenating heterogeneous features may introduce redundant information and semantic inconsistency. Second, high predictive accuracy alone is insufficient for a safety-sensitive application if the model relies on irrelevant background cues rather than morphologically relevant structures. Therefore, an effective mushroom recognition model should coordinate global semantic information with local discriminative features while also providing interpretable evidence for its predictions.

To address these issues, this paper proposes Att-FiLM, an attention-enhanced multi-scale Feature-Wise Linear Modulation network for mushroom image recognition. The proposed architecture employs an asymmetric dual-backbone design in which ConvNeXt-Base provides semantic priors and EfficientNet-B0 extracts task-specific local features. Rather than directly concatenating heterogeneous backbone representations, semantic features are transformed into scale and shift parameters through an attention-enhanced FiLM mechanism and are used to modulate EfficientNet features at Stage 2 and Stage 4. This design provides structured interaction between global semantic context and local morphological information.

The main contributions of this study are summarized as follows:

- An asymmetric dual-backbone framework is developed for mushroom image recognition, using ConvNeXt-Base to provide semantic priors and EfficientNet-B0 to capture local discriminative features.
- An attention-enhanced multi-scale FiLM mechanism is introduced to perform channel-wise modulation at Stage 2 and Stage 4, providing a structured alternative to direct heterogeneous feature concatenation.
- The proposed method is evaluated on both poisonous/edible binary classification and 190-class species-level fine-grained classification tasks. The experimental results demonstrate competitive recognition performance, while the binary classification results also show a reduced false-negative rate for poisonous samples.
- Spatial-response and FiLM-parameter analyses are conducted to examine the decision evidence learned by the model, showing responses associated with morphologically relevant structures such as gills, annuli, volvae, and cap textures.

## 2. Related Work

### 2.1. Fine-Grained Visual Recognition

Fine-grained visual recognition aims to distinguish subordinate categories with small inter-class differences and relatively large intra-class variations. Unlike conventional image classification, the discriminative evidence in fine-grained tasks is often concentrated in subtle local regions, textures, and structural attributes. Early representative approaches, such as bilinear CNNs, modeled interactions between local feature channels to improve fine-grained discrimination [5]. More recent studies have explored feature alignment, progressive feature refinement, multi-scale complementary fusion, dynamic feature interaction, and cross-feature collaboration to enhance local discriminative representation [6,7,8,9,10,11].

Transformer-based architectures have also become increasingly relevant to fine-grained recognition. Vision Transformer (ViT) introduced self-attention-based image representation by modeling relationships among image patches [12]. TransFG further adapted the Transformer architecture to fine-grained visual recognition by selecting discriminative image patches according to attention relationships and introducing contrastive learning to improve separation between visually similar categories [13]. In addition, Swin Transformer constructs hierarchical visual representations through shifted-window self-attention, providing multi-scale feature modeling with reduced computational complexity compared with global self-attention [14]. These developments indicate that both convolutional multi-scale representations and Transformer-based global interaction are relevant to fine-grained recognition. Accordingly, a Swin-T baseline is included in the species-level experiments to provide a representative Transformer-based comparison.

For mushroom recognition, the fine-grained challenge is particularly pronounced because visually similar species may differ only in localized morphological characteristics. Effective recognition therefore requires the model to retain local details while incorporating broader semantic context. The proposed Att-FiLM differs from direct feature fusion approaches by using semantic information from one backbone to condition intermediate features of another backbone through channel-wise modulation.

### 2.2. Mushroom Image Recognition

Deep learning has been increasingly applied to automatic mushroom recognition. Ketwongsa et al. investigated deep learning models for poisonous and edible mushroom classification [15], while Chen et al. studied wild mushroom recognition using an improved Xception-based architecture [16]. Lightweight and detection-oriented approaches have also been explored. For example, PM-YOLO was developed for poisonous mushroom detection by combining lightweight design with contextual and spatial feature calibration [17]. More recently, ensemble learning has been investigated for fungal image classification to improve recognition robustness across visually similar categories [18]. These studies demonstrate the effectiveness of deep visual models for mushroom-related recognition tasks.

However, several issues remain important for natural-scene mushroom recognition. First, field images commonly contain cluttered backgrounds, illumination variation, occlusion, and substantial differences in object scale. Second, morphological distinctions between species may be confined to small structures, making fine-grained feature preservation particularly important. Third, most existing approaches primarily emphasize predictive performance, whereas the relationship between model predictions and morphologically relevant evidence remains less extensively examined. For poisonous mushroom recognition, this issue is especially relevant because false-negative predictions may have more serious practical consequences than ordinary classification errors.

In addition to the binary poisonous/edible task, species-level recognition introduces a substantially more difficult fine-grained setting because a large number of categories must be distinguished using subtle morphological cues. Therefore, this study evaluates Att-FiLM on both binary classification and a 190-class species-level task, allowing the proposed feature modulation strategy to be examined under different levels of classification granularity.

### 2.3. Feature Modulation and Visual Interpretability

Multi-scale and multi-backbone feature fusion is widely used to integrate complementary representations. Conventional concatenation or weighted summation can increase representation capacity, but heterogeneous features may differ considerably in semantic level, channel dimension, and response distribution. Consequently, direct fusion may introduce redundant information or semantic inconsistency. Feature-Wise Linear Modulation (FiLM) provides a conditional feature interaction mechanism in which scale and shift parameters are generated from auxiliary information and applied to intermediate feature channels through affine transformation [19]. This mechanism provides an alternative to directly merging heterogeneous feature vectors and motivates the conditional modulation strategy adopted in Att-FiLM.

Interpretability is also important for image recognition in safety-sensitive scenarios. Class activation mapping and its variants, including CAM, Grad-CAM, and Score-CAM, have been widely used to identify image regions associated with network predictions [20,21,22]. Previous mushroom recognition studies have also explored visual explanations to investigate model attention and discriminative regions [23,24]. In parallel, dimensionality-reduction techniques such as t-SNE and UMAP provide complementary tools for examining the distribution of learned features or internal representations [25,26].

Unlike relying solely on post-hoc spatial visualization, this study analyzes both spatial feature responses and the internal FiLM modulation parameters. This provides two complementary perspectives on model behavior: where discriminative responses occur in the image and how semantic priors alter intermediate feature channels during prediction.

## 3. Methods

### 3.1. Overview of Att-FiLM

**Figure 1:**
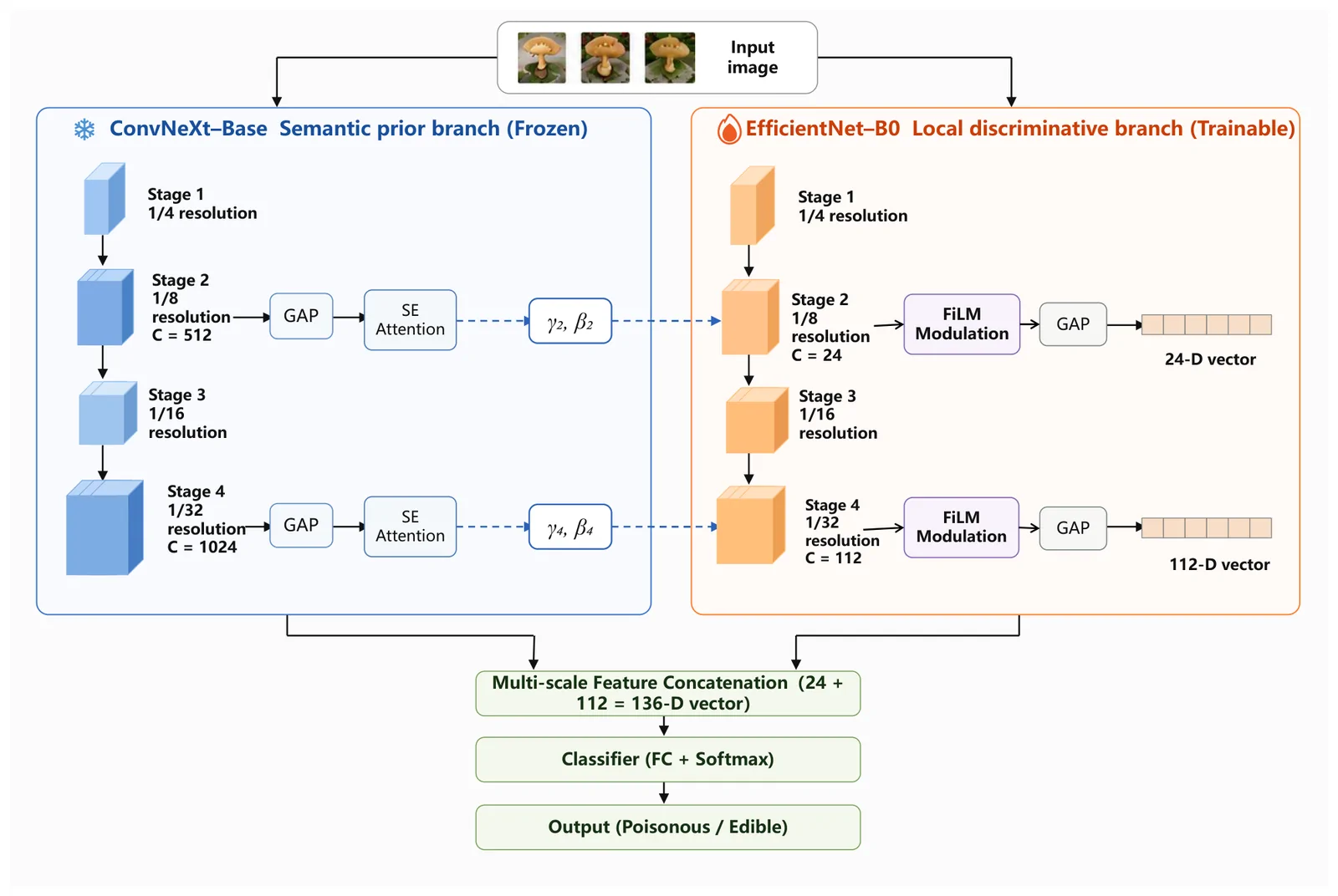
Overall architecture of Att-FiLM. ConvNeXt-Base provides semantic prior features, while EfficientNet-B0 extracts local discriminative features. Semantic features from Stage 2 and Stage 4 are transformed into attention-enhanced FiLM parameters and used to modulate the corresponding EfficientNet-B0 feature maps before multi-scale feature aggregation and classification. Dashed arrows indicate the semantic-conditioning paths through which the generated FiLM parameters are transferred to the corresponding EfficientNet-B0 feature maps. The figure illustrates the default binary-classification configuration in which the ConvNeXt-Base branch is kept frozen.

The proposed Att-FiLM network is designed to coordinate global semantic information and local discriminative features for mushroom image recognition. As illustrated in Figure 1, the architecture consists of three main components: a semantic prior branch based on ConvNeXt-Base [27], a local discriminative branch based on EfficientNet-B0 [28], and an attention-enhanced Feature-Wise Linear Modulation (FiLM) module.

The ConvNeXt-Base branch provides high-level semantic and structural information, whereas the EfficientNet-B0 branch captures task-specific local morphological features, including gills, annuli, volvae, and cap textures. Rather than directly concatenating heterogeneous backbone features, Att-FiLM transforms semantic features into channel-wise scale and shift parameters and uses them to modulate intermediate EfficientNet-B0 features at Stage 2 and Stage 4. This conditional interaction allows semantic priors to guide local feature learning without directly increasing the dimensionality of the fused representation.

### 3.2. Asymmetric Dual-Backbone Feature Extraction

Given an input mushroom image $X\in R^{H\times W\times 3}$, ConvNeXt-Base and EfficientNet-B0 extract semantic and local discriminative features in parallel. Let $G_{l}(\cdot )$ and $E_{l}(\cdot )$ denote the feature extraction functions of ConvNeXt-Base and EfficientNet-B0 at stage *l*, respectively. The multi-scale features are defined as

(1) $$F_{c}^{l}=G_{l}(X;\theta_{c}),\;F_{e}^{l}=E_{l}(X;\theta_{e}),\;l\in L=\left\{2,4\right\}$$

where $F_{c}^{l}$ and $F_{e}^{l}$ denote the feature maps produced by ConvNeXt-Base and EfficientNet-B0 at stage *l*, respectively, while $\theta_{c}$ and $\theta_{e}$ denote their model parameters. In the poisonous/edible binary-classification experiments, the ConvNeXt-Base branch is kept fixed and serves as a stable semantic prior, whereas EfficientNet-B0 is optimized for task-specific feature learning. For the 190-class species-level extension, a staged fine-tuning strategy is adopted to accommodate the more challenging fine-grained recognition setting, as described in Section 4.5.

The two branches play different representational roles. ConvNeXt-Base emphasizes semantic and structural context, whereas EfficientNet-B0 retains lightweight local representations that are suitable for capturing subtle morphological differences. This functional asymmetry provides the basis for subsequent conditional feature modulation.

### 3.3. Multi-Scale Semantic Conditioning

Discriminative mushroom characteristics may appear at different feature scales. Intermediate features retain local textures, edges, and structural details, whereas deeper features contain stronger semantic information. Att-FiLM therefore performs semantic conditioning at Stage 2 and Stage 4 to incorporate complementary information from different representation levels.

For each selected stage *l*, the ConvNeXt feature map $F_{c}^{l}$ is compressed into a semantic vector using global average pooling

(2) $$z_{c,k}^{l}=\mathrm{GAP}(F_{c,k}^{l})=\frac{1}{H_{l}W_{l}}\sum_{i=1}^{H_{l}}\sum_{j=1}^{W_{l}}F_{c,k}^{l}(i,j)\;k=1,2,\ldots ,C_{l}$$

where $H_{l}$, $W_{l}$, and $C_{l}$ denote the height, width, and number of channels of the feature map at stage *l*, respectively. The resulting vector $z_{c}^{l}$ summarizes global semantic information and is subsequently used to generate the FiLM modulation parameters. In contrast, the EfficientNet-B0 feature map $F_{e}^{l}$ retains its spatial organization and serves as the target of semantic modulation.

### 3.4. Attention-Enhanced FiLM Modulation

Not all semantic channels contribute equally to fine-grained mushroom recognition. To emphasize informative semantic responses before generating FiLM parameters, channel attention is applied to the pooled ConvNeXt representation. For stage *l*, the attention weights are computed as

(3) $$a^{l}=\sigma \left(W_{2}^{l}\delta \left(W_{1}^{l}z_{c}^{l}\right)\right)$$

where $W_{1}^{l}$ and $W_{2}^{l}$ are learnable projection matrices, δ(·) denotes the ReLU activation function, and σ(·) denotes the sigmoid activation function.

The semantic representation is refined through element-wise channel reweighting

(4) $${\tilde{z}}_{c}^{l}=a^{l}⊙z_{c}^{l}$$

where ⊙ denotes element-wise multiplication.

The attention-refined semantic representation is then projected into the scale parameter $\gamma^{l}$ and shift parameter $\beta^{l}$

(5) $$\gamma^{l}=W_{\gamma }^{l}{\tilde{z}}_{c}^{l}+b_{\gamma }^{l}\;\beta^{l}=W_{\beta }^{l}{\tilde{z}}_{c}^{l}+b_{\beta }^{l}$$

where $W_{\gamma }^{l}$, $W_{\beta }^{l}$, $b_{\gamma }^{l}$, and $b_{\beta }^{l}$ are learnable projection parameters. The generated vectors are reshaped into broadcastable channel-wise parameters and applied to the corresponding EfficientNet-B0 feature map.

For channel *k*, the FiLM-modulated feature is defined as

(6) $${\tilde{F}}_{e,k}^{l}(i,j)=\gamma_{k}^{l}F_{e,k}^{l}(i,j)+\beta_{k}^{l}\;k=1,2,\ldots ,C_{l}$$

where ${\tilde{F}}_{e,k}^{l}(i,j)$ denotes the modulated response at spatial position (i,j) of channel *k*. The scale parameter $\gamma_{k}^{l}$ controls the contribution of the corresponding local feature channel, whereas $\beta_{k}^{l}$ adjusts its activation offset. In this way, semantic priors can selectively enhance or suppress local discriminative responses without directly concatenating heterogeneous feature maps.

### 3.5. Classification and Training Objective

After modulation at Stage 2 and Stage 4, the two EfficientNet-B0 feature maps are independently pooled and concatenated to construct the final multi-scale representation

(7) $$h=\mathrm{Concat}\left(\mathrm{GAP}\left({\tilde{F}}_{e}^{2}\right),\mathrm{GAP}\left({\tilde{F}}_{e}^{4}\right)\right)$$

where *h* denotes the aggregated representation containing complementary information from the two modulation stages.

The representation is then passed to the classification layer to obtain class probabilities

(8) $$\hat{y}=\mathrm{Softmax}\left(W_{h}h+b_{h}\right)$$

where $W_{h}$ and $b_{h}$ denote the classifier parameters and $\hat{y}$ denotes the predicted class-probability distribution. For poisonous/edible recognition, $\hat{y}$ contains two probabilities, whereas for species-level recognition it contains probabilities over all mushroom species.

For the binary-classification task, model optimization is based on the cross-entropy objective

(9) $$L_{ce}=-\frac{1}{B}\sum_{i=1}^{B}\sum_{c=1}^{C}y_{i,c}\log\left({\hat{y}}_{i,c}\right)$$

where *B* denotes the batch size, *C* denotes the number of classes, $y_{i,c}$ is the ground-truth label indicator, and ${\hat{y}}_{i,c}$ denotes the predicted probability that sample *i* belongs to class *c*.

The optimization strategy is adapted to the difficulty of the two classification tasks. In the binary task, ConvNeXt-Base is kept frozen throughout training, while EfficientNet-B0, the attention modules, the FiLM parameter-generation layers, and the classifier are optimized. For the 190-class species-level task, the two backbone networks are initially frozen and subsequently fine-tuned using a lower learning rate, while the newly introduced modulation and classification layers are optimized with a higher learning rate. The detailed optimization settings for each task are provided in Section 4.

## 4. Experiments and Discussion

To evaluate the effectiveness of Att-FiLM for fine-grained poisonous mushroom image recognition, experiments were conducted from five aspects. First, the overall recognition performance was evaluated on a poisonous/edible binary classification task. Second, Att-FiLM was compared with single-backbone models and a simple dual-backbone concatenation model to examine whether conditional modulation was more effective than direct heterogeneous feature fusion. Third, we conducted ablation experiments to analyze heterogeneous feature fusion, FiLM-based conditional modulation, channel attention, and backbone optimization strategies. Fourth, model complexity and inference efficiency were evaluated in terms of parameters, trainable parameters, FLOPs, inference time, and FPS. Finally, the task was extended to 190-class species-level fine-grained classification to further examine the generalization ability of the proposed method.

### 4.1. Dataset and Experimental Settings

The poisonous/edible binary-classification experiments were conducted using the publicly available *predict poison mushroom by photo* dataset from Kaggle, provided by Stepan Dupliak [29]. The dataset contains 11,703 RGB mushroom images acquired under diverse imaging conditions. According to the current Kaggle data card, no specific license is declared and the license field is listed as “Unknown”. The dataset contains 6937 edible-mushroom images and 4766 poisonous-mushroom images, corresponding to approximately 59.28% and 40.72% of the complete dataset, respectively.

**Figure 2:**
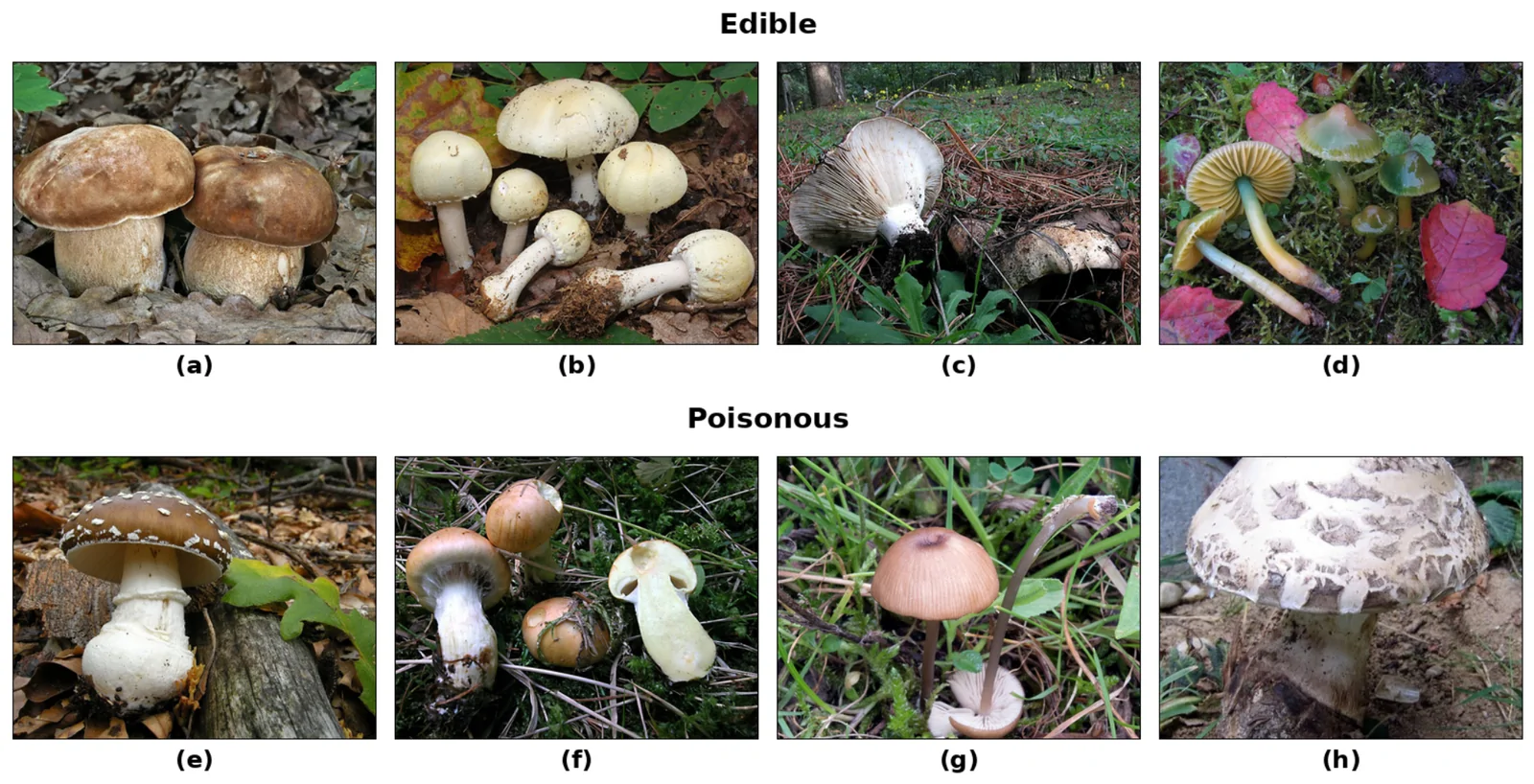
Representative examples from the poisonous/edible mushroom dataset. Panels (**a**–**d**) show edible mushrooms, whereas panels (**e**–**h**) show poisonous mushrooms. The examples illustrate variations in illumination, viewpoint, morphology, and background conditions, as well as visual similarities between the two classes that increase the difficulty of reliable classification in natural scenes.

The images exhibit substantial variations in illumination, viewpoint, object scale, vegetation occlusion, and background composition. In addition, edible and poisonous mushrooms can present similar cap shapes, colors, and overall appearances, whereas discriminative information is often concentrated in subtle local structures such as gills, stipes, and cap textures. Representative examples from the two classes are shown in Figure 2. These characteristics make reliable poisonous/edible recognition challenging under natural-scene conditions.

The dataset used in the reported experiments was organized into a training subset and a fixed validation subset. The training subset contains 10,532 images, including 6243 edible and 4289 poisonous samples, while the validation subset contains 1171 images, including 694 edible and 477 poisonous samples. The detailed class distribution is summarized in Table 1. No separate independent test subset was used in the retained experimental protocol; therefore, the binary-classification performance reported in this study corresponds to the fixed validation subset.

For the binary-classification task, training images were processed using RandomResizedCrop to obtain inputs of 224×224 pixels, followed by random horizontal flipping, color jittering, and normalization using the ImageNet mean and standard deviation. During validation, images were first resized to 256 pixels and then center-cropped to 224×224 pixels. ImageNet pre-trained weights were used for the backbone networks where applicable.

All binary-classification models were trained for 50 epochs with a batch size of 32. AdamW was used as the optimizer with an initial learning rate of $1\times 10^{-3}$ and a weight decay of 0.01. The learning rate was adjusted using cosine annealing over the training process, and cross-entropy loss was used as the optimization objective. For Att-FiLM, the ConvNeXt-Base semantic-prior branch remained frozen during binary-classification training, while the EfficientNet-B0 branch, attention modules, FiLM parameter-generation layers, and classifier were optimized.

To reduce the influence of random initialization and data shuffling, the main binary-classification experiments were independently repeated using five random seeds: 42, 123, 456, 789, and 101. The random states of Python, NumPy, and PyTorch were fixed for each run, and deterministic CuDNN computation was enabled. The experiments were implemented using Python 3.8.20, NumPy 1.15.1, and PyTorch 2.4.1 with CUDA 12.1. The reported results are presented as mean ± standard deviation over the five runs. In addition, a paired *t*-test based on matched random seeds was used to assess the statistical significance of the performance difference between Att-FiLM and the principal EfficientNet-B0 baseline.

The binary-classification evaluation metrics include Accuracy, Precision, Recall, F1-score, ROC-AUC, and false-negative rate (FNR). Poisonous mushrooms are defined as the positive class. In particular, Recall and FNR are emphasized because a false-negative prediction corresponds to a poisonous mushroom being incorrectly classified as edible, which represents a more critical error in safety-sensitive recognition scenarios.

The experiments were implemented using PyTorch and executed on NVIDIA GeForce RTX 3090 GPU hardware. For inference-efficiency evaluation, the latency was measured using a batch size of 1 with a 224×224 input. Before timing, 50 forward passes were performed for GPU warm-up. The average inference latency was then calculated over 200 forward passes with CUDA synchronization to ensure reliable timing measurements.

### 4.2. Comparison with Baseline Models

To evaluate the effectiveness of Att-FiLM, three representative baseline configurations were considered: EfficientNet-B0, ConvNeXt-Base, and a simple heterogeneous dual-backbone model denoted as Concat. EfficientNet-B0 serves as the principal lightweight convolutional baseline and evaluates the discriminative capability of a single local-feature backbone. ConvNeXt-Base evaluates the recognition performance of the semantic backbone when used independently. Concat directly concatenates features extracted from the two heterogeneous backbones and therefore provides a reference for determining whether simple feature aggregation is sufficient to exploit their complementary information. All models were evaluated using the same training and validation protocol described in Section 4.1. The results over five independent runs are summarized in Table 2.

As shown in Table 2, EfficientNet-B0 achieves an Accuracy of 94.01%, an F1-score of 0.9253, and an ROC-AUC of 0.9840. These results indicate that the lightweight convolutional backbone is capable of learning useful local morphological features from mushroom images. However, its Recall is 0.9111 and its FNR reaches 0.0889, indicating that some poisonous samples remain difficult to distinguish when only a single local-feature extraction pathway is used.

ConvNeXt-Base achieves substantially lower performance than EfficientNet-B0 in the current setting. This result does not imply that ConvNeXt is intrinsically unsuitable for visual recognition; rather, it reflects the role assigned to this branch in Att-FiLM. In the binary configuration, ConvNeXt-Base is used as a frozen semantic-prior extractor rather than as a fully fine-tuned task-specific classifier. Consequently, its independently extracted high-level semantic representation is less effective for directly distinguishing subtle local mushroom morphology. The result supports the asymmetric design of Att-FiLM, in which ConvNeXt provides semantic conditioning while EfficientNet-B0 remains responsible for learning task-specific local discriminative features.

The Concat model achieves an Accuracy of 93.27% and an F1-score of 0.9162, both lower than those of EfficientNet-B0. Therefore, merely adding a second backbone does not lead to improved recognition performance. Direct concatenation treats heterogeneous features as equally compatible representations despite differences in their semantic levels, channel distributions, and feature scales. Such unconstrained aggregation may introduce redundant or conflicting information and consequently weaken the discriminative features learned by the local branch.

Att-FiLM achieves the best performance among the evaluated configurations, with an Accuracy of 95.58±0.28%, an F1-score of 0.9455±0.0034, and an ROC-AUC of 0.9903±0.0009. Compared with EfficientNet-B0, Att-FiLM improves the mean Accuracy by approximately 1.57 percentage points and increases the F1-score from 0.9253 to 0.9455. In addition, the FNR decreases from 0.0889 to 0.0629. Since poisonous mushrooms are treated as the positive class, this reduction indicates fewer poisonous samples being incorrectly predicted as edible.

To further examine whether the observed improvement was attributable to random variation across training runs, paired *t*-tests were conducted using the results obtained from the five matched random seeds. For Accuracy, Att-FiLM significantly outperformed EfficientNet-B0 (t(4)=12.52, p<0.001). A consistent result was obtained for the F1-score (t(4)=13.11, p<0.001). These results provide statistical support for the performance improvement observed across the repeated experiments, although the statistical analysis should be interpreted in the context of the limited number of independent runs.

The improvement over both the single-backbone and direct-concatenation baselines indicates that the benefit of Att-FiLM is primarily associated with structured cross-backbone interaction rather than simply increased model capacity. By transforming the semantic representation from ConvNeXt-Base into sample-dependent scale and shift parameters, the proposed method regulates EfficientNet-B0 features at multiple levels without directly mixing the two heterogeneous feature spaces. This mechanism preserves local morphological information while incorporating global semantic guidance.

Previous mushroom-recognition studies have demonstrated the effectiveness of convolutional classification, transfer learning, ensemble learning, and object-detection-based approaches [15,16,17,18]. However, differences in datasets, species composition, task definitions, and evaluation protocols prevent a direct numerical comparison between those reported results and the present binary-classification experiment. In contrast to methods that primarily focus on improving the classification backbone or detection pipeline, Att-FiLM specifically addresses heterogeneous global–local feature interaction through conditional modulation. The present results therefore complement existing mushroom-recognition studies by showing that explicitly controlling cross-backbone information transfer can improve fine-grained poisonous/edible recognition while reducing false-negative predictions.

### 4.3. Ablation Study

To examine the contribution of the main design choices in Att-FiLM, ablation experiments were conducted on the poisonous/edible binary-classification task. The evaluated variants focus on heterogeneous feature fusion, FiLM-based conditional modulation, channel-attention-guided semantic conditioning, and backbone optimization strategies. All variants used the same data split and evaluation protocol. The results are summarized in Table 3.

Variant A uses EfficientNet-B0 alone and therefore represents the local-discriminative baseline. Variant B introduces the ConvNeXt-Base branch but directly concatenates heterogeneous features. Its Accuracy decreases from 94.01% to 93.27%, while its F1-score decreases from 0.9253 to 0.9162. This result indicates that simply increasing feature dimensionality does not guarantee complementary information. Differences in semantic level, feature scale, and channel distribution between the two backbones can limit the effectiveness of direct concatenation.

Replacing direct concatenation with FiLM-based conditional modulation produces a clear improvement. Variant C reaches an Accuracy of 95.10%, an F1-score of 0.9395, and an ROC-AUC of 0.9888. Instead of directly mixing heterogeneous representations, FiLM transforms the semantic information extracted by ConvNeXt-Base into sample-dependent scale and shift parameters that regulate the EfficientNet feature channels. This result supports conditional modulation as a more structured interaction mechanism between the semantic and discriminative branches.

Adding channel attention to the FiLM configuration produces the complete Att-FiLM model, which further improves Accuracy from 95.10% to 95.58%, F1-score from 0.9395 to 0.9455, and ROC-AUC from 0.9888 to 0.9903. The additional gain is moderate, indicating that channel attention acts primarily as a refinement component. It reweights the semantic representation before the generation of FiLM parameters so that more informative channels can exert greater influence on the subsequent modulation process.

Variant I applies attention-based semantic reweighting without the complete FiLM scale-and-shift transformation. Although its Accuracy remains at 94.01%, its F1-score and ROC-AUC increase to 0.9383 and 0.9894, respectively. This suggests that semantic attention alone can improve class discrimination, but it does not provide the same overall benefit as combining attention with conditional feature modulation.

Variant H jointly optimizes the two backbone networks and obtains an Accuracy of 93.66% and an F1-score of 0.9211. Under the current dataset scale, joint optimization therefore does not improve performance over the asymmetric configuration. A plausible explanation is that simultaneously updating the high-capacity semantic branch increases optimization difficulty and the risk of overfitting, whereas keeping ConvNeXt-Base fixed provides a stable semantic prior for conditioning the lighter EfficientNet-B0 branch.

Overall, the ablation results indicate that the performance of Att-FiLM cannot be attributed simply to the use of an additional backbone. The largest improvement is obtained when direct heterogeneous feature concatenation is replaced by FiLM-based conditional modulation, while channel attention provides an additional refinement. These results support the combined use of asymmetric semantic conditioning, conditional feature modulation, and attention-guided channel selection.

### 4.4. Model Complexity and Inference Efficiency

In addition to recognition performance, computational complexity and inference efficiency are important considerations for practical image-recognition systems. Therefore, the evaluated configurations were compared in terms of total parameters, trainable parameters, FLOPs, inference latency, and throughput. The latency measurements followed the protocol described in Section 4.1, using a batch size of 1, a 224×224 input, 50 warm-up forward passes, and 200 synchronized forward passes on an NVIDIA GeForce RTX 3090 GPU (NVIDIA Corporation, Santa Clara, CA, USA). The results are summarized in Table 4.

As shown in Table 4, EfficientNet-B0 has the lowest computational cost, requiring only 0.828 GFLOPs and achieving an inference latency of 4.85 ms per image. Att-FiLM is computationally heavier because it incorporates the ConvNeXt-Base semantic branch. Its total parameter count reaches 94.30M and its computational cost is 31.585 GFLOPs. However, most of these parameters belong to the frozen semantic-prior branch. Under the default binary-classification configuration, only 5.71M parameters are trainable.

The additional modulation components themselves introduce little inference overhead relative to the corresponding dual-backbone configurations. The Concat model requires 28.25 ms per image, whereas FiLM and Att-FiLM require 28.32 and 28.42 ms, respectively. Thus, introducing FiLM and channel attention increases latency by only 0.17 ms relative to direct concatenation. Att-FiLM maintains a throughput of 35.18 FPS under the adopted measurement protocol. These results indicate that the main computational cost originates from the dual-backbone architecture, whereas the proposed conditional modulation mechanism adds only a small incremental inference cost.

Training-resource consumption was also recorded for different ConvNeXt optimization strategies. The default frozen Att-FiLM configuration required approximately 80.89 min of training time and 4266.89 MB of recorded GPU memory. Partial fine-tuning required 99.60 min and 4669.32 MB, whereas full fine-tuning required 155.23 min and 14,939.80 MB, respectively. Although partial fine-tuning produced a slightly higher Accuracy of 95.71% and an F1-score of 0.9470, the improvement over the frozen configuration was small relative to the additional training cost. The frozen semantic-prior configuration therefore provides a more favorable balance between recognition performance and training-resource requirements.

Although Att-FiLM is substantially more computationally demanding than EfficientNet-B0, its design is not intended to minimize the total parameter count. Instead, the asymmetric freezing strategy limits the number of optimized parameters while retaining the semantic representation capability of a stronger backbone. This trade-off should be considered when the model is applied in resource-constrained or latency-sensitive scenarios.

### 4.5. Extension to 190-Class Species-Level Classification

The preceding experiments focus on poisonous/edible binary classification. To further evaluate whether Att-FiLM can retain its discriminative capability under a substantially more complex category system, the task was extended to species-level fine-grained recognition involving 190 mushroom categories. Compared with the binary setting, this task contains substantially more categories, fewer samples per category, greater intra-class appearance variation, and stronger inter-class morphological similarity.

The species-level dataset was constructed by integrating three public mushroom-image collections recorded in the original project materials as mushrooms, MO106, and fungi. We identified the first collection as the Mushrooms dataset provided by Derek Kuno-Williams on Kaggle [30] and the second as the MO106 dataset released by the Image Processing Research Laboratory of the University of Pannonia [31,32]. The third collection was recorded only as fungi in the retained project materials. Because source-specific metadata were not preserved after dataset merging and preprocessing, we were unable to reliably recover its original uploader and repository address.

During preprocessing, images from the source collections were merged and reorganized according to species labels. YOLOv10 [33] was employed only as an offline preprocessing tool to localize mushroom regions and generate cropped candidate images. It is not part of the Att-FiLM architecture or the inference pipeline evaluated in this study. Following automatic cropping, more than 1200 images were manually sampled and inspected to assess cropping quality and label consistency.

Detector-level performance records from the preprocessing stage were not retained because YOLOv10 was not an experimental target of the original classification study. Consequently, detector mAP, precision, and recall are not reported. Automatic localization may nevertheless introduce residual errors, such as partial target truncation, inaccurate bounding regions, or excessive background inclusion. Although manual inspection was used to reduce these problems, remaining cropping errors constitute a limitation of the current dataset-construction procedure.

After preprocessing and quality control, the retained dataset contains 9363 images from 190 species. The number of images per species ranges from 40 to 60, with an average of approximately 49.3 images per class. The final experimental directory contains a training subset of 7432 images and a fixed validation subset of 1931 images, corresponding to approximately 79.4% and 20.6% of the complete dataset, respectively. All 190 species are represented in both subsets. The training subset contains 32–48 images per class, whereas the validation subset contains 8–12 images per class. The dataset statistics are summarized in Table 5.

During the original preprocessing and merging procedure, the retained dataset was reorganized according to species labels and did not preserve separate per-image source identifiers. Therefore, the exact post-filtering number of images contributed by each of the three original repositories cannot be reliably reconstructed from the retained experimental data. Similarly, a separate quantitative log for systematic near-duplicate removal was not retained. We therefore do not report per-source post-filtering contributions or quantitative deduplication statistics that cannot be reconstructed from the retained experimental records.

For the 190-class experiment, the input resolution was increased to 384×384 pixels to preserve fine-grained morphological information. Training images were processed using RandomResizedCrop, random horizontal flipping, RandAugment with two operations and a magnitude of 9, and ImageNet normalization. Validation images were resized to 400 pixels and center-cropped to 384×384 pixels. A batch size of 32 was used.

All models were initialized with ImageNet pre-trained weights where applicable and trained for 100 epochs. AdamW was used with a weight decay of 0.01. Class-frequency weighting and label smoothing of 0.1 were applied to the cross-entropy objective. For Att-FiLM, the backbone networks were frozen during the first five epochs while the newly introduced layers were optimized using a learning rate of $1\times 10^{-3}$. From the sixth epoch onward, the backbones were unfrozen and optimized using a lower learning rate of $1\times 10^{-5}$. Cosine annealing was applied throughout training, and exponential moving averaging with a decay factor of 0.999 was used for evaluation.

To provide a stronger comparison with modern visual architectures, Swin-T [14] was additionally evaluated as a representative hierarchical Transformer baseline using the same 190-class data split and training protocol. The comparative results are reported in Table 6.

As shown in Table 6, EfficientNet-B0 and ResNet-50 [34] achieve Top-1 Accuracies of 86.75% and 88.42%, respectively, whereas ConvNeXt-Base reaches 92.15%. The results indicate that stronger semantic representation becomes increasingly important as the number of visually similar categories increases.

Swin-T provides a substantially stronger comparison than the conventional CNN baselines, achieving a Top-1 Accuracy of 93.53%, a Top-5 Accuracy of 98.86%, and a Macro-F1 of 0.9335. Att-FiLM achieves the highest Top-1 Accuracy of 93.63% and the highest Macro-F1 of 0.9347, while matching Swin-T in Top-5 Accuracy. The margin between Att-FiLM and Swin-T is small, indicating that the hierarchical Transformer baseline is highly competitive on this species-level task. Nevertheless, Att-FiLM retains a slight advantage under the same evaluation setting while employing an explicitly structured global-to-local conditional modulation mechanism.

Compared with EfficientNet-B0, Att-FiLM improves Top-1 Accuracy by 6.88 percentage points and Macro-F1 by 0.0712. Compared with ConvNeXt-Base, the improvements are 1.48 percentage points in Top-1 Accuracy and 0.0162 in Macro-F1. These results suggest that the proposed method is not restricted to poisonous/edible binary classification and remains effective when the task is extended to a substantially larger set of visually similar species.

The species-level results should nevertheless be interpreted separately from the binary-classification results. The 190-class experiment uses a higher input resolution, stronger data augmentation, staged backbone fine-tuning, and a different classification objective. It therefore serves as an additional evaluation of the representational capability of Att-FiLM under a more difficult fine-grained setting rather than as a direct continuation of the binary experimental protocol.

## 5. Interpretability Analysis

For safety-sensitive mushroom recognition, classification performance alone does not reveal which visual evidence contributes to model predictions. A model may obtain high accuracy while partially exploiting background patterns, illumination conditions, or other incidental image characteristics. Therefore, this section examines the internal behavior of Att-FiLM from three complementary perspectives: spatial responses of decision-relevant channels, class-dependent distributions of FiLM modulation parameters, and the discriminative behavior of deep modulation channels. The purpose is to determine whether the learned representations are associated with mushroom morphology and to clarify how the conditional modulation mechanism behaves across different semantic levels.

### 5.1. Core-Channel Spatial Response

**Figure 3:**
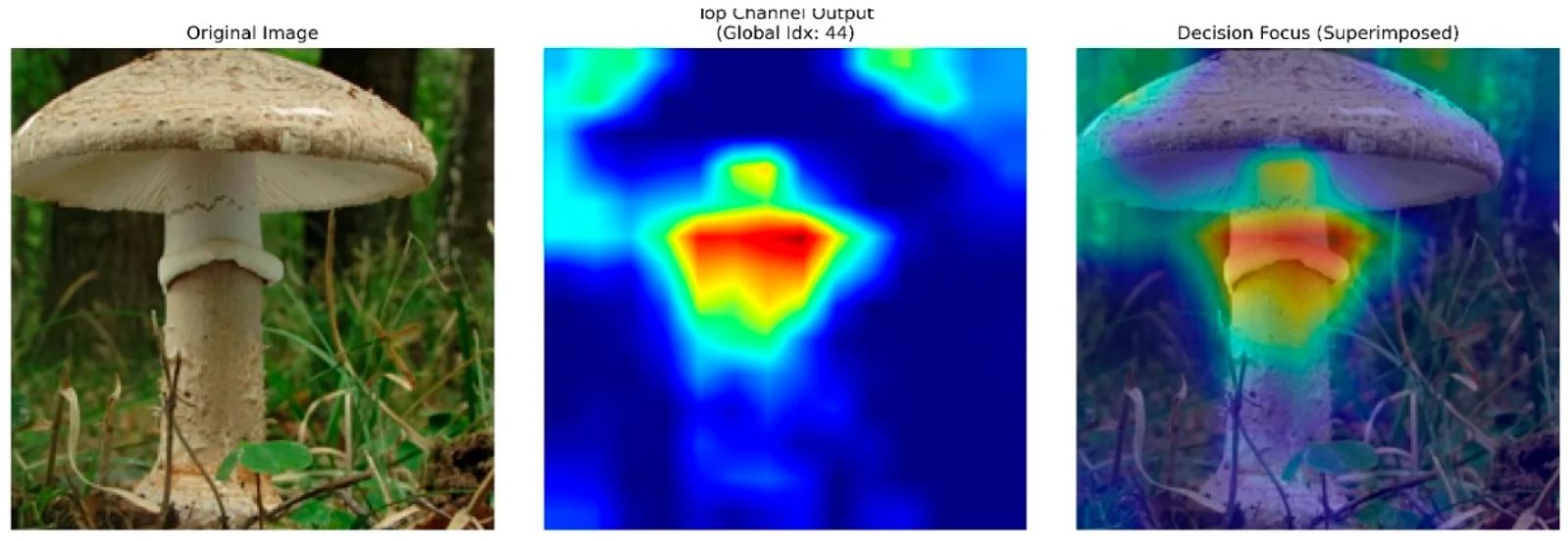
Core-channel spatial response of Att-FiLM for a representative poisonous mushroom image. The visualization presents the original image, the response map of a decision-relevant channel, and the corresponding response overlaid on the original image. Warmer colors (red/yellow) indicate stronger responses, while cooler colors (blue) indicate weaker responses in the spatial response map.

To examine the spatial evidence associated with Att-FiLM predictions, we selected a decision-relevant channel according to its contribution to the classification logit for a representative poisonous mushroom sample. We then projected its activation response onto the input-image space and overlaid it on the original image. This analysis provides a qualitative view of the image region associated with a highly contributing internal feature. The representative result is shown in Figure 3.

As shown in Figure 3, the spatial response visualization illustrates the activation distribution of a decision-relevant feature channel generated by Att-FiLM for a representative poisonous mushroom image. The strongest response is concentrated on the mushroom body, particularly around morphological structures near the gills, annulus, and stipe, while relatively weaker responses appear in the surrounding background regions. This indicates that the examined decision-relevant feature is spatially associated with discriminative mushroom morphology rather than being dominated by irrelevant background information. Nevertheless, this response map should be interpreted as qualitative evidence of feature association rather than proof of biological causality.

### 5.2. Distribution of FiLM Modulation Parameters

The FiLM module regulates EfficientNet feature channels through sample-dependent scale parameters γ and shift parameters β. Among these parameters, γ directly controls the magnitude and direction of channel-wise feature scaling and therefore provides a useful representation for examining conditional modulation behavior.

**Figure 4:**
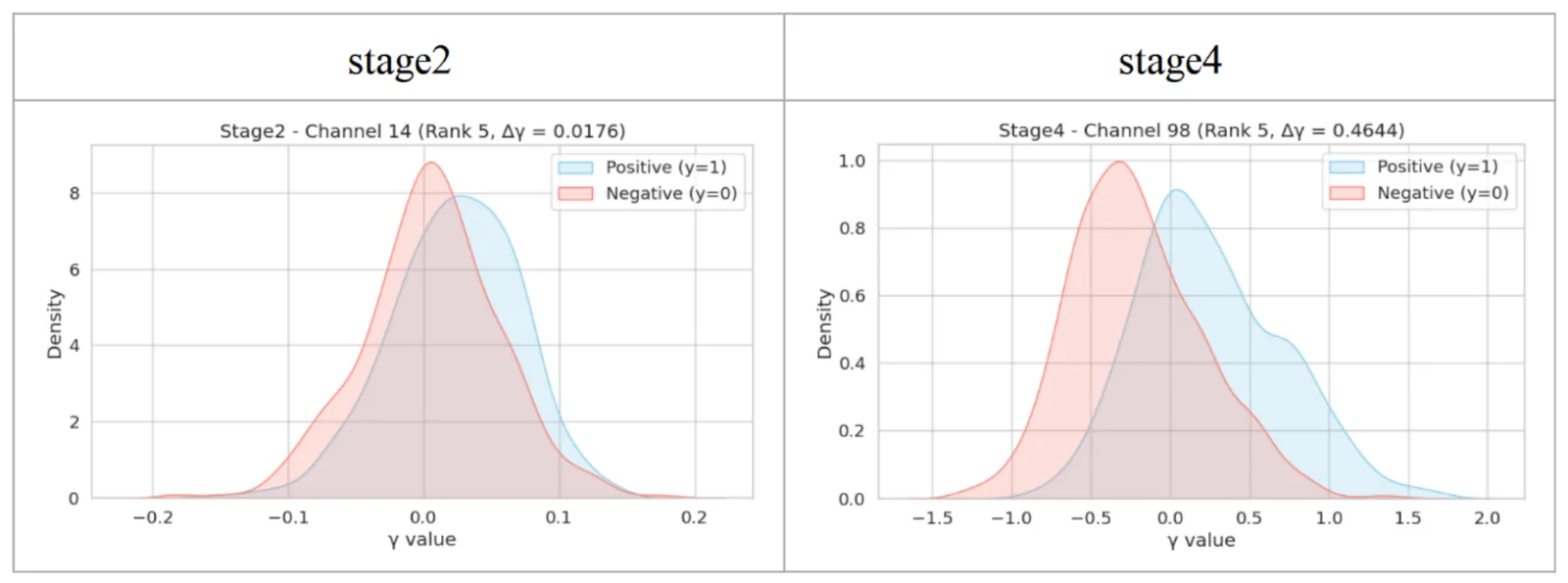
Class-conditional distributions of representative FiLM scale parameters γ at Stage 2 and Stage 4. Kernel density estimation curves compare the modulation responses of poisonous and edible mushroom samples for one representative channel at each stage.

To investigate whether the modulation exhibits class-dependent behavior, we examined representative channels at Stage 2 and Stage 4 and used kernel density estimation to visualize the γ values obtained from poisonous and edible samples. The results are shown in Figure 4.

As shown in Figure 4, the representative Stage 2 channel exhibits substantial overlap between the poisonous and edible distributions, suggesting relatively weak class-dependent modulation at the shallower feature level. In contrast, the representative Stage 4 channel shows a more pronounced distribution shift between the two classes, indicating stronger class-conditioned modulation at the deeper semantic level.

We attribute this difference to the hierarchical structure of the network. Stage 2 retains relatively general local information, whereas Stage 4 contains representations with stronger semantic abstraction. The observed distributions therefore suggest that shallow and deep feature stages exhibit different degrees of class-dependent modulation.

### 5.3. Discriminative Contribution of Deep Modulation Channels

Because Stage 4 exhibits clearer class-dependent separation, its modulation channels were further examined quantitatively. For each Stage 4 γ channel, the difference between the mean modulation values of poisonous and edible samples was calculated. The ten channels with the largest absolute class-dependent differences were selected, as summarized in Table 7. Poisonous and edible samples are denoted by y=1 and y=0, respectively.

Table 7 shows that the Stage 4 modulation behavior is not uniform across channels. For example, Channels 34, 43, and 101 have negative mean γ values for poisonous samples but positive values for edible samples. In contrast, Channels 97, 98, and 20 show positive mean modulation for poisonous samples and negative values for edible samples. Thus, different channels exhibit opposite class-conditioned modulation patterns.

These results indicate that the semantic branch does not simply increase or decrease all EfficientNet channels simultaneously. Instead, the generated FiLM parameters selectively regulate individual feature channels according to the input. Channels that are enhanced for one class may be suppressed for the other, producing a class-dependent reorganization of the discriminative representation. This observation is consistent with the purpose of FiLM in Att-FiLM, namely to convert global semantic information into channel-wise control signals for the local-discriminative branch.

Taken together, Figure 3 and Figure 4 and Table 7 provide complementary evidence regarding the internal behavior of Att-FiLM. The spatial analysis shows that decision-relevant responses in the examined images frequently overlap with mushroom morphological structures. The parameter-distribution analysis shows that deeper FiLM channels exhibit stronger differences between poisonous and edible samples than shallower channels. The channel-level statistics further demonstrate that these differences include both class-dependent enhancement and suppression rather than uniform feature amplification.

Overall, the interpretability analysis suggests that Att-FiLM learns structured, sample-dependent modulation behavior that is associated with fine-grained mushroom morphology. These observations improve the transparency of the proposed feature-interaction mechanism and provide qualitative and quantitative evidence for understanding how semantic conditioning influences the discriminative branch. However, the presented analyses remain diagnostic interpretations of model behavior and should not be regarded as biological or causal proof of mushroom toxicity.

## 6. Conclusions

This study proposed Att-FiLM, an attention-enhanced multi-scale Feature-Wise Linear Modulation network for fine-grained poisonous mushroom recognition. The model combines a ConvNeXt-Base semantic-prior branch with an EfficientNet-B0 discriminative branch and uses FiLM-based channel-wise modulation to achieve structured interaction between global semantic information and local morphological features.

On the poisonous/edible binary-classification task, Att-FiLM achieved an Accuracy of 95.58%, an F1-score of 0.9455, and an ROC-AUC of 0.9903 on the fixed validation subset. Compared with EfficientNet-B0, the mean Accuracy improved by approximately 1.57 percentage points, while the false-negative rate decreased from 0.0889 to 0.0629. On the 190-class species-level task, Att-FiLM achieved a Top-1 Accuracy of 93.63% and a Macro-F1 of 0.9347, remaining competitive with the Swin-T Transformer baseline. Ablation and interpretability analyses further indicated that FiLM-based conditional modulation is the main contributor to the performance gain and that decision-relevant responses are frequently associated with morphological structures such as gills, annuli, volvae, and cap textures.

The current study still has several limitations, including the absence of independent external test sets and incomplete provenance records for the merged 190-class dataset. In addition, YOLOv10 was used as an offline preprocessing tool without retained detector-level evaluation records. Future work will focus on external validation across broader geographic and environmental conditions, more traceable dataset construction, lightweight deployment, and integration of ecological information with visual features. 

## Figures and Tables

**Table 1:** Class distribution of the poisonous/edible mushroom dataset.

Class	Training	Validation	Total
Edible	6243	694	6937
Poisonous	4289	477	4766
Total	10,532	1171	11,703

**Table 2:** Performance comparison of different models on the fixed validation subset of the poisonous/edible binary-classification task. Results are reported as mean ± standard deviation over five independent runs. The best value for each metric is shown in bold.

Model	Accuracy (%)	Precision	Recall	F1-score	ROC-AUC	FNR
EfficientNet-B0	94.01±0.35	0.9399±0.0056	0.9111±0.0066	0.9253±0.0044	0.9840±0.0014	0.0889±0.0066
ConvNeXt-Base	84.08±0.26	0.8056±0.0023	0.8029±0.0058	0.8043±0.0036	0.9192±0.0006	0.1964±0.0060
Concat	93.27±0.41	0.9284±0.0071	0.9042±0.0082	0.9162±0.0055	0.9826±0.0021	0.0958±0.0082
Att-FiLM	95.58±0.28	0.9497±0.0051	0.9413±0.0023	0.9455±0.0034	0.9903±0.0009	0.0629±0.0063

**Table 3:** Ablation study of the key components of Att-FiLM on the binary-classification task. The best value for each metric is shown in bold.

Model	Accuracy (%)	F1-Score	ROC-AUC
A (Baseline)	94.01	0.9253	0.9840
B (Concat)	93.27	0.9162	0.9826
C (FiLM)	95.10	0.9395	0.9888
D (Att-FiLM)	**95.58**	**0.9455**	**0.9903**
H (Joint)	93.66	0.9211	0.9828
I (Attention-only)	94.01	0.9383	0.9894

**Table 4:** Model complexity and inference-efficiency comparison on the binary-classification task.

Model	Params (M)	Trainable (M)	FLOPs (G)	Latency (ms/Image)	FPS
EfficientNet-B0	4.01	4.01	0.828	4.85	206.10
ConvNeXt-Base (Frozen)	87.57	0.00	30.745	26.50	37.72
Concat	93.88	5.29	31.573	28.25	35.47
FiLM	94.13	5.54	31.580	28.32	35.34
Att-FiLM	94.30	5.71	31.585	28.42	35.18

**Table 5:** Statistics of the 190-class species-level mushroom dataset.

Statistic	Value
Number of classes	190
Total images	9363
Training images	7432 (79.4%)
Validation images	1931 (20.6%)
Minimum images per class	40
Maximum images per class	60
Mean images per class	49.3
Training images per class	32–48
Validation images per class	8–12

**Table 6:** Performance comparison on the 190-class species-level fine-grained classification task. The best value for each metric is shown in bold; tied best values are both shown in bold.

Model	Top-1 Acc. (%)	Top-5 Acc. (%)	Macro-Precision	Macro-Recall	Macro-F1
EfficientNet-B0	86.75	94.12	0.8720	0.8580	0.8635
ResNet-50	88.42	95.30	0.8875	0.8750	0.8805
ConvNeXt-Base	92.15	97.92	0.9250	0.9168	0.9185
Swin-T	93.53	**98.86**	0.9389	0.9343	0.9335
Att-FiLM	**93.63**	**98.86**	**0.9426**	**0.9354**	**0.9347**

**Table 7:** Stage 4 FiLM scale channels with the ten largest differences in mean modulation values between poisonous and edible samples.

Channel Index	|Δγ|	E[γ|y=1]	E[γ|y=0]
34	0.5035	−0.1119	0.3916
43	0.5020	−0.1159	0.3861
101	0.4890	−0.1281	0.3610
97	0.4690	0.4266	−0.0425
98	0.4644	0.2500	−0.2144
20	0.4473	0.3015	−0.1459
50	0.4402	−0.2713	0.1689
8	0.4253	−0.0227	0.4025
17	0.4238	−0.3158	0.1080
10	0.4068	−0.2912	0.1156

## Data Availability

The datasets used in this study were derived from publicly available mushroom image datasets, including the Mushrooms dataset available on Kaggle and the MO106 mushroom image dataset released by the Image Processing Research Laboratory of the University of Pannonia. The corresponding repository information and URLs are provided in references [30,32]. The processed datasets, dataset split information, and experimental records generated during this study are available from the corresponding author upon reasonable request.

## Abbreviations

The following abbreviations are used in this manuscript:

Att-FiLM	Attention-enhanced Feature-Wise Linear Modulation
FiLM	Feature-Wise Linear Modulation
CNN	Convolutional Neural Network
GAP	Global Average Pooling
FLOPs	Floating Point Operations
FPS	Frames Per Second
FNR	False Negative Rate
ROC-AUC	Area Under the Receiver Operating Characteristic Curve

## References

[1] Li W., Pires S.M., Liu Z., Liang J., Wang Y., Chen W., Liu C., Liu J., Han H., Fu P. (2021). Mushroom Poisoning Outbreaks—China, 2010–2020. China CDC Wkly..

[2] Li H., Zhang Y., Zhang H., Zhou J., Li Z., Yin Y., He Q., Jiang S., Zhang Y., Yuan Y. (2025). Mushroom Poisoning Outbreaks—China, 2024. China CDC Wkly..

[3] Yao Q., Wu Z., Zhong J., Yu C., Li H., Hu Q., He J., Du J., Sun C. (2023). A Network System for the Prevention and Treatment of Mushroom Poisoning in Chuxiong Autonomous Prefecture, Yunnan Province, China: Implementation and Assessment. BMC Public Health.

[4] Amirul S.N., Mohd Sabri N., Tan G.J., Redwan N.A., Zhang Z. (2025). Convolutional Neural Network Based Mobile Application for Poisonous Mushroom Detection. ESTEEM Acad. J..

[5] Lin T.-Y., RoyChowdhury A., Maji S. Bilinear CNN Models for Fine-Grained Visual Recognition. Proceedings of the IEEE International Conference on Computer Vision (ICCV).

[6] Wu Q., Song T., Fan S., Chen Z., Jin K., Zhou H. (2024). Feature Alignment via Mutual Mapping for Few-Shot Fine-Grained Visual Classification. Image Vis. Comput..

[7] Ma Z.-X., Chen Z.-D., Zheng T., Luo X., Jia Z., Xu X.-S. (2025). Few-Shot Fine-Grained Image Classification with Progressively Feature Refinement and Continuous Relationship Modeling. Proc. AAAI Conf. Artif. Intell..

[8] Lei J., Yang X., Yang S. (2022). Multiscale Progressive Complementary Fusion Network for Fine-Grained Visual Classification. IEEE Access.

[9] Cheng Y., Li B., Jia P. (2025). MDF-Net: Multilayer Feature Dynamic Interactive Fusion Network for Few-Shot Fine-Grained Image Classification. Vis. Comput..

[10] Qiu S., Yang W., Yang M. Hybrid Feature Collaborative Reconstruction Network for Few-Shot Fine-Grained Image Classification. Proceedings of the 2025 IEEE International Conference on Acoustics, Speech and Signal Processing (ICASSP).

[11] Yang S., Yang X., Wu J., Feng B. (2024). Significant Feature Suppression and Cross-Feature Fusion Networks for Fine-Grained Visual Classification. Sci. Rep..

[12] Dosovitskiy A., Beyer L., Kolesnikov A., Weissenborn D., Zhai X., Unterthiner T., Dehghani M., Minderer M., Heigold G., Gelly S. An Image Is Worth 16x16 Words: Transformers for Image Recognition at Scale. Proceedings of the 9th International Conference on Learning Representations (ICLR).

[13] He J., Chen J.-N., Liu S., Kortylewski A., Yang C., Bai Y., Wang C. (2022). TransFG: A Transformer Architecture for Fine-Grained Recognition. Proc. AAAI Conf. Artif. Intell..

[14] Liu Z., Lin Y., Cao Y., Hu H., Wei Y., Zhang Z., Lin S., Guo B. Swin Transformer: Hierarchical Vision Transformer Using Shifted Windows. Proceedings of the IEEE/CVF International Conference on Computer Vision (ICCV).

[15] Ketwongsa W., Boonlue S., Kokaew U. (2022). A New Deep Learning Model for the Classification of Poisonous and Edible Mushrooms Based on Improved AlexNet Convolutional Neural Network. Appl. Sci..

[16] Chen D., Azragul, Yin P., Lu Y., Li S. (2021). Research on Identification of Wild Mushroom Species Based on Improved Xception Transfer Learning. Laser Optoelectron. Prog..

[17] Wei Z., Wang J., You H., Ji R., Wang F., Shi L., Yu H. (2025). A Lightweight Context-Aware Framework for Toxic Mushroom Detection in Complex Ecological Environments. Ecol. Inform..

[18] Kumru E., Korkmaz A.F., Ekinci F., Aydoğan A., Güzel M.S., Akata I. (2025). Deep Ensemble Learning and Explainable AI for Multi-Class Classification of Earthstar Fungal Species. Biology.

[19] Perez E., Strub F., de Vries H., Dumoulin V., Courville A. (2018). FiLM: Visual Reasoning with a General Conditioning Layer. Proc. AAAI Conf. Artif. Intell..

[20] Zhou B., Khosla A., Lapedriza A., Oliva A., Torralba A. Learning Deep Features for Discriminative Localization. Proceedings of the IEEE Conference on Computer Vision and Pattern Recognition (CVPR).

[21] Selvaraju R.R., Cogswell M., Das A., Vedantam R., Parikh D., Batra D. Grad-CAM: Visual Explanations from Deep Networks via Gradient-Based Localization. Proceedings of the IEEE International Conference on Computer Vision (ICCV).

[22] Wang H., Wang Z., Du M., Yang F., Zhang Z., Ding S., Mardziel P., Hu X. Score-CAM: Score-Weighted Visual Explanations for Convolutional Neural Networks. Proceedings of the IEEE/CVF Conference on Computer Vision and Pattern Recognition Workshops (CVPRW).

[23] Korkmaz A.F., Ekinci F., Altaş Ş., Kumru E., Güzel M.S., Akata I. (2025). A Deep Learning and Explainable AI-Based Approach for the Classification of Discomycetes Species. Biology.

[24] Özbay E., Özbay F.A., Gharehchopogh F.S. (2024). Visualization and Classification of Mushroom Species with Multi-Feature Fusion of Metaheuristics-Based Convolutional Neural Network Model. Appl. Soft Comput..

[25] van der Maaten L., Hinton G. (2008). Visualizing Data Using t-SNE. J. Mach. Learn. Res..

[26] McInnes L., Healy J., Saul N., Großberger L. (2018). UMAP: Uniform Manifold Approximation and Projection. J. Open Source Softw..

[27] Liu Z., Mao H., Wu C.-Y., Feichtenhofer C., Darrell T., Xie S. A ConvNet for the 2020s. Proceedings of the IEEE/CVF Conference on Computer Vision and Pattern Recognition (CVPR).

[28] Tan M., Le Q.V. EfficientNet: Rethinking Model Scaling for Convolutional Neural Networks. Proceedings of the 36th International Conference on Machine Learning (ICML).

[29] Dupliak S. Predict Poison Mushroom by Photo. Kaggle. https://www.kaggle.com/datasets/stepandupliak/predict-poison-mushroom-by-photo.

[30] Kuno-Williams D. Mushrooms. Kaggle. https://www.kaggle.com/datasets/derekkunowilliams/mushrooms.

[31] Kiss N., Czúni L. Mushroom Image Classification with CNNs: A Case-Study of Different Learning Strategies. Proceedings of the 2021 12th International Symposium on Image and Signal Processing and Analysis (ISPA).

[32] Image Processing Research Laboratory, University of Pannonia MO106 Mushroom Image Dataset. https://keplab.mik.uni-pannon.hu/en/mo106eng.

[33] Wang A., Chen H., Liu L., Chen K., Lin Z., Han J., Ding G. (2024). YOLOv10: Real-Time End-to-End Object Detection. Adv. Neural Inf. Process. Syst..

[34] He K., Zhang X., Ren S., Sun J. Deep Residual Learning for Image Recognition. Proceedings of the IEEE Conference on Computer Vision and Pattern Recognition (CVPR).

